# The Prevalence and Cause(s) of Burnout Among Applied Psychologists: A Systematic Review

**DOI:** 10.3389/fpsyg.2018.01897

**Published:** 2018-10-16

**Authors:** Hannah M. McCormack, Tadhg E. MacIntyre, Deirdre O'Shea, Matthew P. Herring, Mark J. Campbell

**Affiliations:** ^1^Physical Education and Sport Science Department, University of Limerick, Limerick, Ireland; ^2^Health Research Institute, University of Limerick, Limerick, Ireland; ^3^Kemmy Business School, University of Limerick, Limerick, Ireland

**Keywords:** mental health, well-being, burnout, stress, psychologists, coping, systematic review

## Abstract

**Purpose:** Burnout has been shown to develop due to chronic stress or distress, which has negative implications for both physical and mental health and well-being. Burnout research originated in the “caring-professions.” However, there is a paucity of research which has focused specifically on how job demands, resources and personal characteristics affect burnout among practitioner psychologists.

**Methods:** This PRISMA review (Moher et al., 2009) involved searches of key databases (i.e., Web of Knowledge, SCOPUS and Google Scholar) for articles published prior to 1st January, 2017. Articles concerning the prevalence and cause(s) of burnout in applied psychologists, that were published in the English language were included. Both quantitative and qualitative investigative studies were included in the review. The Crowe Critical Appraisal Tool (CCAT; Crowe, 2013) was used to appraise the quality of each paper included in this review. An inductive content analysis approach (Thomas, 2006) was subsequently conducted in order to identify the developing themes from the data.

**Results:** The systematic review comprised 29 papers. The most commonly cited dimension of burnout by applied psychologists was emotional exhaustion (34.48% of papers). Atheoretical approaches were common among the published articles on burnout among applied psychologists. Workload and work setting are the most common job demands and factors that contribute to burnout among applied psychologists, with the resources and personal characteristics of research are age and experience, and sex the most commonly focused upon within the literature.

**Conclusions:** The results of the current review offers evidence that burnout is a concern for those working in the delivery of psychological interventions. Emotional exhaustion is the most commonly reported dimension of burnout, with job and personal characteristics and resources also playing important roles in the development of burnout in the mental health care profession. Finally, tentative recommendations for those within the field of applied psychology.

## Introduction

### Rationale

Burnout is defined as the end state of long-term chronic stress (Maslach, 2003), and is a syndrome represented by three dimensions; mental fatigue or emotional exhaustion, negative feelings and perceptions about the people one works with or depersonalization, and a decrease in feelings of personal accomplishment (Maslach and Jackson, 1981). Burnout is considered by many as a “work-related mental-health impairment” (Awa et al., 2010, p. 184), and is often correlated with anxiety and depression (Morse et al., 2012). Not only can burnout be personally distressing (Freudenberger, 1975), it may also manifest itself in many physical and mental health related issues (Maslach et al., 2001). Physical symptoms present as fatigue, exhaustion and somatization, and it is also linked to social withdrawal, the inability to regulate the expression of emotions (Gorgievski and Hobfoll, 2008); absenteeism, (Ahola et al., 2008); lowered morale and reduced efficiency and performance (Taris, 2006). Some conceptualizations of burnout argue that it is unidimensional in nature, pertaining only to exhaustion (Pines and Aronson, 1988; Kristensen et al., 2005; Shirom and Melamed, 2005) and thus there are measures of burnout that examine this dimension only. However, Maslach's (1982, 1993) three-dimensional model of burnout and the *Maslach Burnout Inventory* are considered the “gold standard” in burnout research (Schutte et al., 2000, p. 53).

Early research into the phenomenon of burnout focused on employees in health-care services, as these were the “occupations in which the goal is to provide aid and service to people in need and which can therefore be characterized by emotional and interpersonal stressors” (Bakker et al., 2014, p. 390). Burnout can negatively influence the quality of one's work and therefore the standard of care provided to clients (Rupert et al., 2015), and is “hypothesized to produce a generalized negative outlook toward self and others” (Paris and Hoge, 2010, p. 521). Thus, burnout is not only harmful to the employee themselves (e.g., the psychologist in this case), but may also have secondary harmful effects on clients and patients (Rupert et al., 2015). In particular, the depersonalization dimension of burnout can lead to the emotional distancing or disengagement of a psychologist from their clients (Maslach and Jackson, 1981). It is this duality of concern, for both the individual and those within their care, which justifies further investigation and review of burnout among applied psychologists.

According to Maslach and Leiter (2005) the sources of burnout include one or more of the following: workload (too much work, not enough resources); control (micromanagement, lack of influence, accountability without power); reward (not enough pay, acknowledgment, or satisfaction); community (isolation, conflict, disrespect); fairness (discrimination, favoritism); and values (ethical conflicts, meaningless tasks). Burnout can result from a mismatch between the individual and the work environment. Tasks that require sustained physical, emotional, or cognitive effort equate to job demands (Demerouti et al., 2001). If job demands remain persistently high, an individual may become burnt out, experiencing chronic fatigue, even distancing themselves psychologically from their work (Bakker et al., 2000).

The Jobs Demands Resources Model (JD-R, Demerouti et al., 2001) and the Conservation of Resources model (Hobfoll, 1989; Hobfoll and Freedy, 1993; COR, Halbesleben et al., 2014) are two commonly used theories within burnout research, they posit that burnout predominantly occurs because of untreated or unresolved chronic stress and distress (Rupert et al., 2015). Both theories also suggest job and personal resources can contribute to the prevention or reduction of burnout (Rupert et al., 2015). Resources are defined as, “anything perceived by the individual to help attain his or her goals” (Halbesleben et al., 2014 p. 1338). Key job resources include opportunities for professional development, supervision and feedback (Halbesleben et al., 2014); autonomy (Hobfoll, 1989; a supportive supervisor or supervisory team Hobfoll, 1989, 2002; and regular positive feedback (Maslach and Leiter, 2005). Personal resources, which further buffer against burnout can include self-efficacy, resilience and a comprehensive recovery process (Halbesleben et al., 2014). Both job and personal resources go directly toward influencing an individual's motivation or engagement at work and can buffer the demands of a person's job (Rupert et al., 2015). COR (Hobfoll, 1989) explains the advantage of seeking and maintaining resources in times of stress, and is based on the supposition that people will strive to retain, protect and build their resources. Once an individual possesses positive resources, it becomes easier for them to obtain more resources, resulting in a gain spirals (Hobfoll, 2001; Hakanen et al., 2008; Halbesleben and Wheeler, 2015). Current research into burnout among psychologists does not utilize these theoretical frameworks (Rupert et al., 2015). Providing psychological support and the administration of psychotherapy are examples of job demands that are associated with feelings of responsibility toward the client, maintaining strong and healthy relationships with clients, dealing with other people's problems and emotional concerns, and managing clients who are challenging or difficult (Farber and Heifetz, 1981; Deutsch, 1984; Hellman and Morrison, 1987; Stevanovic and Rupert, 2004) which are unique to applied psychologists and allied mental health professionals.

By recognizing the demands, resources, and work and personal characteristics that contribute to, or alleviate, burnout among applied psychologists we are able to better understand the process behind the development and synthesis of burnout among this population. Applying these theories, will not only reveal a clearer picture of the current research but will also pave the way for future investigation.

Psychologists and allied mental health professionals are also subject to a number of work related health impairments, including compassion fatigue (Figley, 2002) secondary traumatization (Canfield, 2005) and vicarious traumatization (Dunkley and Whelan, 2006). Burnout, itself has been associated with depression both within the field of psychology (Pope and Tabachnick, 1994; Gilroy et al., 2002) and within other professions (Hakanen and Schaufeli, 2012). It has also been shown to mediate between stress and depression (Ahola et al., 2009), with clinicians also report lower feelings of safety with increased emotional exhaustion (Welp et al., 2015).

While causing risk to themselves, high job demands and potential consequential burnout could inadvertently affect the clients of the psychologist through impaired professional functioning, coupled with reduced competence (Rupert et al., 2015). When an individual withdraws from their role they will not strive to do their best and may instead do the bare minimum to get by (Maslach, 2003), which could lead to obvious consequences when other people's well-being is at stake. Burnout in psychologists can also raise ethical concerns (Rupert et al., 2015), stemming from the inability to continue practicing competently. At some stage in their careers, psychologists will be faced with scenarios that push the boundaries and it is imperative that they continue to practice ethically throughout this time (Koocher and Keith-Spiegel, 2008). According the APA code of ethics (2016), a psychologist must practice within the boundaries of their competence (2.01) and they must continually engage in the development and maintenance of their competence, (2.03) they must be aware of any personal problems that may negatively competence and take appropriate action to deal with them (2.06). For example, the Canadian code of ethics for psychologists explicitly states that psychologists would “engage in self-care activities that help avoid conditions (e.g.,m burnout, addictions) that could result in impaired judgement and interfere with their ability to benefit and not harm others” (Koocher and Keith-Spiegel, 2008, p. 574). Therefore, it is clear that burnout represents a potential personal problem that may negatively influence competence and it is one which psychologists are thus ethically bound to address.

### Objectives

Evidence into the causes and effects of burnout in general working populations has accumulated over the years (Bakker et al., 2006; Borgogni et al., 2012); however, information regarding the burnout of applied psychologists has not kept pace with that of other professions. A 2016 report from the British Psychological Society based on a survey by New Savoy found that 70% of psychotherapists found their job stressful, with a quarter considering that they have a long-term chronic condition, 46% reported depression. The purpose of this review was to synthesize research evidence regarding burnout among applied psychologists. To achieve this goal, we reviewed the extant literature focusing on the prevalence of burnout in the wider field of applied psychology, including psychologists from across the various sub-disciplines. It was predicted that this study could enhance our understanding of burnout in applied psychology in general.

### Research question

Based on the information presented above, the aim of this study was to examine the prevalence and cause(s) of burnout among applied psychologists and allied mental health professionals, focusing on resources and their influence on burnout. The knowledge gained from this review will initiate further investigation into the burnout amongst applied psychologists consulting in all disciplines.

## Methods

### Search strategy

This systematic review was conducted in accordance with the PRISMA statement guidelines (Moher et al., 2009). Criteria set out to identify and eventually include papers in this review, databases were searched, duplicate papers eliminated with biases accounted for, results were then synthesized and presented in compliance with PRISMA guidelines (Moher et al., 2009). This review focused on the extant literature on stress and burnout of applied psychologists. Studies were restricted to those published in English. Articles published on the topic of the prevalence and cause(s) of burnout and stress in applied psychologists prior to 1st January 2017 were located using the following databases: Web of Knowledge, SCOPUS and Google Scholar. The search strategy included a combination of the following terms: “burnout” AND (psychologist^*^OR “mental health professional” ^*^) AND (stress^*^OR “emotional exhaustion”^*^ OR pressure OR cop^*^ OR manage^*^ *OR “well-being” OR “mental health”)*. Reference lists from the retrieved articles were manually reviewed to identify any additional potential inclusions.

### Study selection

**Figure 1:**
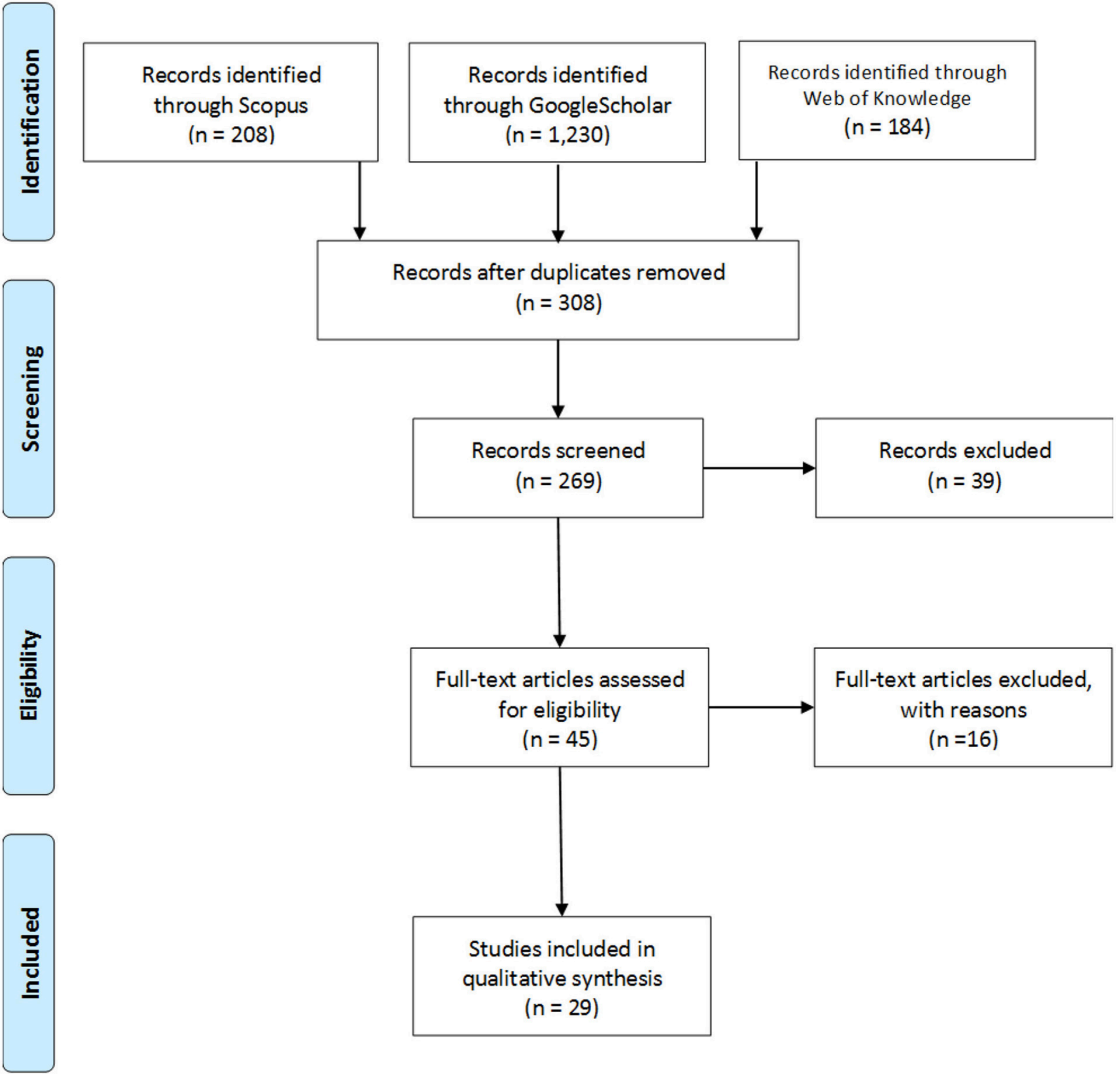
PRISMA flow diagram. This figure illustrates the steps taken in conducting the systematic review.

The review included articles that met the following criteria: (i) peer-reviewed publication available in English (foreign language papers were excluded due to the cost and time it would take to translate them; Arksey and O'Malley, 2005); (ii) the sample had to include those offering psychological services, but who did not have medical training; (iii) measured burnout and/or its constituent dimensions; (iv) publications with original data (i.e., meta-analytic reviews, narrative reviews, and unpublished theses etc. were not included). Studies were eliminated if they examined nurses, doctors, administrators or other health professionals as their main cohort, despite these professionals working in a mental health setting. Initial searches returned 308 non-duplicate articles. Abstracts and titles were scrutinized in order to assess whether these citations met inclusion criteria. Thirty-nine articles were excluded due to not being original research articles. Forty-five articles were examined in full, with 16 of these excluded during the full text screening. As a result, 29 articles were included in this review. See Figure 1 for the PRISMA flow diagram.

### Data extraction and analysis

The characteristics of all of the papers included in the review were inputted into a table. Included papers were analyzed under the following headings: (i) sample size and professional specialization, (ii) research instruments used, (iii) theoretical framework, (iv) predictors studied, (iv) the dimensions of burnout that were specifically examined, and (v) the main findings and results of each paper. The CCAT procedure (Crowe, 2013) was applied to all papers, assigning each a score for quality out of a possible maximum of 40 (see Table 1). Each paper was scrutinized on its quality of preliminaries (title, abstract), introduction, design, sampling, data collection, ethical matters, results and, discussion (Crowe, 2013). An inductive content analysis approach (Thomas, 2006) was employed in order to identify the themes emerging from the data. The majority of studies took a quantitative approach to their investigation. Two papers took a qualitative approach with a semi-structured interview methodology. Overall, burnout appeared to be a major concern for psychologists. Data from the review shows the most commonly cited dimension of burnout was emotional exhaustion (34.48% of papers).

**Table 1:** Study Characteristics and CCAT scores.

**Author(s)**	**Country**	**Design**	**Sample/specialty**	**Aim of paper**	**Theoretical Framework**	**Burnout dimensions measured**	**Predictors of burnout studied**	**Results**	**CCAT Quality score**
Acker, 2012	USA	1	460 mental health service providers (social workers, psychologists and case managers)	Compare relationships in workplace conditions with role stress, burnout and intent to quit.	**2**	EE	Demographic variables. Work setting and work variables. Career satisfaction.	More than half (56%) of participants reported moderate to high EE, almost three quarters (73%) reported high role stress (RS), and half (50%) reported intention to leave the role. RS significantly predicted burnout. EE significantly predicted the intent to quit, over and above the effect of RS. Those working with patients with severe mental illness report higher levels of burnout and stress. EE is a mediating varibale rather than outcome varibale of burnout.	25
Ackerley et al., 1988	USA	1	562/ Licensed psychologists	Examining the correlates of burnout along with the instance of burnout in national sample.	1	EE, DP, PA	Demographic information. Case type. Work setting and variables.	Licensed psychologists have higher levels of burnout that other mental health workers. EE was the highest rated dimension of burnout. Age negatively related to burnout. Those in private practice experience less EE, DP and more PA than those in agency settings. PP workers less likely to experience burnout or it's correlates. No gender difference in experienced burnout. Psychologist-client relationship influences burnout.	31
Ballenger-Browning et al., 2011	USA	1	97 Civilians and active duty mental health providers in the US military. Psychologists made up the majority of participants (31.5%)	Assess levels and predictors of burnout among mental health providers in a military setting.	1	EE, DP, PA	Providers demographics, social support and situational variables.	27 (27.8%) providers scored in the high level of emotional exhaustion, 18 (18.6%) had high depersonalization scores, and 4 (4.1%) reported a low level of personal accomplishment. Gender, profession and hours worked predicted burnout. Case type influences DP. Employment, experience and support increased PA. High case load decreased PA.	26
Ben-Zur and Michael, 2007	Israel	1	249/Social workers (55.8%), Psychs (20.5%) and Nurses (23.7%) all female.	Compare burnout and related characteristic between social workers, psychologists and nurses and assess effectiveness of appraisal, support and coping.	**3**	EE, DP, PA	Age, number of hours working, and origin.	High burnout is negatively correlated with support at work, challenge/control appraisals, and problem-focused coping. At the same time it is positively correlated to stress/load and emotion-focused coping. Social support is mediated by appraisal and coping	24
Boccio et al., 2016	USA	1	291 school psychologists	Role conflict, administrative pressure and their influence on burnout.	1	EE, DP, PA	Experience with administrative pressure to act unethically or illegally.	High instance of administrative pressure to act unethically and/or illegally. EE was the most highly experienced characteristic of burnout. Administrative power positive related to increased EE, decreased PA, decreased job satisfaction and increased intent to quit.	27
Carrola et al., 2016	USA	1 and/or 2	86 correctional counselors with a minimum masters levels qualification (professional counselors, 64%; licensed psychologists, 11.6%; social worker, 5.8%; 17.4% were not licensed; 1.2% did not specify).	Use of CBI among correctional counselors. Compare burnout rates as related to gender and workplace.	1	EE, DP,PA. Incompetence, negative work environment, deterioration in personal life.	Gender and experience	Higher burnout was experienced by those who work in maximum security environments. Case type correlated with burnout experienced. Gender and work setting also influenced type of burnout experienced.	24
Devilly et al., 2009	Australia	1 and/or 2	152 mental health workers: (125 or 82%) psychologists, (15 or 9.8%) psychotherapists, (6 or 3.9%) clinical social workers, 1 nurse, 1 psychiatrist and 4 other (all engaging in clinical therapeutic work)	Assess STS, VT and burnout among Australian mental health professionals in clinical practice.	**4**	PB, WB, CB	Satisfaction with work. Demographic information. Exposure to trauma.	Burnout contributes to therapists distress more highly than Secondary traumatic stress (STS) and Vicarious trauma (VT). Exposure to trauma patients did not influence burnout experienced. Both VT and burnout contribute significantly to affective distress. Burnout explains more variance in affective distress than VT.	27
Di Benedetto and Swadling, 2014	Australia	1 and/or 2	167/ registered psychologists	Investigate burnout in Australian psychologists, work-setting and years of experience.	1	PB, WB, CB	Work -setting (private vs. public), length of experience, mindfulness.	Burnout did not differ between psychologists dependent on work-setting. The longer someone had worked, the less burnt out they would be. Mindfulness was negatively related to burnout.	30
Dreison et al., 2016	USA	1 and/or 2	358 staff from mental health agencies (Psychology was highest cited discipline. Social work, counseling, nursing, addictions, business and psychiatry)	To identify factors that might protect against burnout.	**4, 5**	EE, DP, PA	Age, education and discipline	Psychologists had significantly higher levels of emotional exhaustion. Education was sig. related to lower DP and lower PA. Men reported higher levels of PA. Age and length of time in the field were not sig. related to any burnout dimensions. Resources interact differently with the different dimensions of burnout.	30
D'Souza et al., 2011	Australia	2	87/ Clinical psychologists	Impact of personality on stress.	1	PB, WB, CB	Years of experience, work variables, access to peer support, client presenting problems, theoretical orientation.	Perfectionism and stress are related to burnout. Those who are highly stressed are more likely to experience different dimensions of burnout. Bunrout is a reflection of continued exposure to stress.	26
Emery et al., 2009	Australia	1	190/ Clinical Psychologists	Examine relationship between burnout, demographics and workplace variants.	1	OS, EE, DP, PA.	Age, gender, professional training, years of professional experience, work-status, client type, work setting, annual income, living arrangement.	Distress exacerbated burnout. Perfectionistic tendencies, therapeutic control contributed to stress. High levels of personal resources detract from EE and PA. Higher levels of EE were significantly associated with being female, working primarily for the government, having less personal resources, and endorsing more therapist beliefs related to control. Personal factors were strongest predictors of EE and PA.	29
Garcia et al., 2016	USA	2	137 (doctoral-level psychologists, psychology residents and interns, master-level counselors, both master- and doctoral-level social workers)	Examine predictors of burnout and intent to leave.	1	EE, DP, PA	Demographics, theoretical orientation, education and experience. Exposure to trauma. Workplace characteristics.	High levels of exhaustion and cynicism were reported, with low PA. Demographic variables not significant for burnout High EE impacted the quality of the service provided. Lack of control influenced exhaustion. Trauma characteristics not related to burnout. Malingering patients increased feelings of cynicism.	26
Hardiman and Simmonds, 2013	Australia	1	89 clinicians, counselors and psychotherapists	Relationship between spiritual well-being and burnout.	1	EE, DP, PA	Demographic information. Exposure to trauma.	Low levels of burnout reported. EE still came out as strongest indicator of burnout. EE scores weakly and negatively related to ratings of severity of client trauma. Age related to burnout.	26
Malinowski, 2013	USA	1	133/psychotherapists	Investigate the relationship between different types of humor and burnout.	**6,7**	EE, DP, PA	Work setting, age, number of years experience after being licensed, average hours worked per week.	Adaptive types of humor used twice as much as maladaptive humor. If looking at the severity levels of burnout as conceptualized by Maslach et al. (1996) therapists who experience EE were between low and moderate levels of severity of burnout. Therapists who experienced DP and decreased PA were on the low level of severity of burnout.	25
McCormack et al., 2015	International	3	30 Applied sport psychologists	Impact of social support on burnout and work engagement.	**8**	Qualitative investigation into the impact of social support and the causes of burnout.	Types of social support	All participants were able to recall easily, moments in their career when they felt burnt out. Often during high pressure situations with increased responsibility and/or high stakes. Burnout was experienced at a lower level than those who credited work based peer support as their main source of social support.	27
Mills and Huebner, 1998	USA	4	225 at T1: 173 at T2/School psychologists	Prevalence and antecedents of burnout in school psychologists.	**9**	EE, DP, PA	Personality, demographics, number of schools, years of experience, job location, number of colleagues, student ratio.	40% reported high levels of EE, 10.2% report high DP, and 18.7% reported low PA. Demographic info did not correlate with burnout. Nuroticism correlated all three burnout dimensions. EE sig. correlation with extraversion, agreeableness and conscientiousness. Depersonalization was related to agreeableness and reduced personal accomplishment was related to extraversion.	31
Proctor and Steadman, 2003	USA	1	31 in house/32 traditional school psychologists (63 total)	Work setting and it's influence on Burnout	1	General burnout	Demographics, job satisfaction, burnout, perceived effectiveness, preference of employment setting and activities.	In-house psychologists report higher job satisfaction and lower burnout. They also report perceived effectiveness. Looking at burnout alone and specifically between items, there are no differences between groups and only the conglomerate score shows a difference. Those serving a single school compared to those who serve multiple schools experience lower levels of burnout. However, overall burnout does not seem to be an issue for the population	27
Puig et al., 2012	USA	2	129 mental health professionals [mental health counselors (29%), psychologists (14.7%), counselor education (8.5%), marriage and family therapy (7%), social work (1.6%), rehabilitation counseling (0.8%) other disciplines (38%)]	Burnout in relation to personal wellness.	1	Exhaustion, incompetence, negative work environment, devaluing client and, deterioration in personal life.	Burnout and wellness	Exhaustion had the highest mean score from the CBI. Devaluing clients had the lowest mean score. The highest means score on the wellness question went to the social self-subscale, with the coping self-receiving the lowest mean score. Job burnout is negatively related to an individuals exercise and nutrition.	29
Rosenberg and Pace, 2006	USA	1	116/ Marriage and family therapists	Predictors and prevalence of burnout.	1	EE, DP, PA	Personal Characteristics. Goals and expectations. Demographics. Professional development. Work setting and work variables.	Some differences in demographics. Females reported lower levels of DP. Masters level had slightly higher levels of PA than doctoral-level. No difference between participants who engaged in professional development or professional support than those who did not. Work setting did influence dimensions of burnout experienced.	23
Rupert and Kent, 2007	USA	1	595/ practicing psychologists	Determine factors that relate to different levels of burnout.	**10, 8**	EE, DP, PA	Gender, work setting, work variables, resources, coping strategies.	Respondents matched the average or middle range of EE and DP for this population, and the lower range of PA. Age was significantly related to burnout. Gender and work-setting correlated to burnout. Men experience greater DP than women. Workload positively related to burnout. Personal resources have a role in preventing burnout. Solo practitioners reported less support.	30
Rupert and Morgan, 2005	USA	1	571/ practicing psychologists	Work setting and it's influence on Burnout. Resources	1	EE, DP, PA	Demographic information, work setting and variables, degree, and theoretical orientation.	Work setting and gender correlated with burnout. Age negatively related to burnout. Workload positively related to burnout.	29
Rupert et al., 2012	USA	1	595/practicing psychologists	Determine career satisfaction in practicing psychologists	1	EE, DP. PA	Career satisfaction. Demographic information, experience, work setting and variables, theoretical orientation, supervision.	Majority of practicing psychologists are satisfied with their work. Control has emerged as a resource related to lower levels of burnout.	25
Rupert et al., 2009	USA	1	487/421 practicing psychologists (those who were living with spouse/partner/children)	Determine difference in work-family conflict and it's contribution to burnout.	**8**	EE, DP, PA (MBI-HSS)/Control, Over involvement, Support, Negative clientele (PBI-R)	Family life, Gender, Work setting	Age is related to burnout. No sig difference between gender and work settings in relation to burnout. Control is negatively related to EE and DP while positively related to PA. Gender differences exist in family life and responsibility of care and exhaustion.	28
Rzeszutek and Schier, 2014	Poland	1	200 Gestalt and cognitive behavioral therapists	Severity of burnout in relation to temperament traits and social support.	**11**	Exhaustion and disengagement from work.	Burnout, temperament and aspects of social support.	No significant difference between Gestalt therapists and CBT therapists. Perceived social support and briskness were both negatively associated with burnout. Perseveration proved to be a significant positive predictor of burnout symptoms in the whole group of therapists	26
Senter et al., 2010	USA	1	203/Correctional psychologists (CR), Veteran's affairs (VA), Counseling Centers (CC), Public Psychiatric Hospital (PPH)	Burnout in correctional psychologists vs. other public sector psychologists.	1	EE, DP, PA	Job satisfaction, life satisfaction.	Correctional facility (CR) psychologists experience higher levels of burnout and report lower levels of job satisfaction. Greater professional identity predicted lower levels of burnout.	29
Sim et al., 2016	USA	3	14 staff psychologists at colleges and university counseling centers.	Experience in thriving, burnout and coping of psychologists in university counseling centers.	**4**	Qualitative comparison of ECPs vs. LCPs	Post-doctoral experience.	Recognition for achievement was typically identified as important by both ECPs and LCPs. Work setting correlated with thriving. Work variables correlated to thriving. Client improvement also contributed to thriving. Burnout was related to challenge, Non-clinical tasks and crisis work. Challenges in professional relationships contributed to burnout. Loneliness and isolation was only reported as a burnout factor by ECPs and not by LCPs Participants used interpersonal support for coping. Self-care, cognitive coping-strategies, behavioral strategies (creating boundaries and adjusting work schedules). Personal therapy only reported by ECPs.	31
Steel et al., 2015	UK	2	116 High Intensity Therapists (HITs) and Psychological Wellbeing Practitioners (PWPs).	Investigate levels and predictors of three burnout dimensions among therapists in IAPT services	1	EE, DP, PA	Predictors chosen from the GMB (General model of burnout): causal factors “demands” and lacking “resources.” Demographics, basic client data.	High levels of EE reported. Low levels of DP reported. Psychological job demands predict EE. Age and psychological job demands predict DP. Training significanlty predicted PA. Resources and feelings of in session flow predicted PA. Demographic variables and case type did not predict burnout. High demands and lack of autonomy predict EE.	30
Viehl and Dispenza, 2015	USA	2	189 mental health practitioners (licensed individuals e.g., psychologists 66.67%, and certified professionals 15.87%). Final sample size of 150.	Differences in coping and burnout among sexual identified minority MHP.	1	Exhaustion, incompetence, negative work environment, devaluing client and, deterioration in personal life.	Gender, sexual orientation.	Male sexual minority related to increased burnout. Sexual-minority identified MHPs engaged in less emotion-focused coping when compared to heterosexual-identified MHPs. Women engaged in more problem-focused coping than men. Sexual minority men in this sample are experiencing more exhaustion, frustration, stress, and not feeling effective as counselors when compared to heterosexual MHPs.	30
Vredenburgh et al., 1999	USA	1	521/counseling psychologists who had PhDs	Extent of burnout and relationships between work-setting, demographics and work-setting variables.	**12**	EE, DP, PA	Demographics, work setting and variables.	Reports of moderately low to medium level burnout. Practice setting has implications for burnout: lower level of PA and DP. Private practice lead to lower levels of burnout in general, probably due to autonomy and income. Client load is positively associated with PA but not related to EE or DP. There is an inverse relationship between age and burnout. Males have greater levels of DP than females.	29

*Study design: 1, Cross sectional survey on Paper; 2, Cross sectional survey via web; 3, semi structured interview; 4, 2-time prospective study*.

*Theoretical Framework: 1, N/A; 2, Role Stress Model; 3, Cognitive model of stress and coping; 4, JD-R; 5, SDT; 6, Theory of multidimensional humor; 7, Maslachs theory of job burnout; 8, COR; 9, Transactional model of burnout, personality and situational stressors; 10, Structural model of burnout; 11, Regulatory theory of temperament; 12, General Model of Burnout*.

*Bold numbers indicate papers which used a theoretical framework*.

## Results

This review sought to examine relevant research regarding the burnout of applied psychologists. We now focus on the emergent themes from all the papers included in this review. Starting with emotional exhaustion as the most prevalent dimension of psychotherapist burnout and subsequently exploring other factors that influence burnout in this population.

### Study selection and quality

Table 1 details the key characteristics of all studies used in the review. Participants included applied psychologists (specialization unknown); clinical psychologists, counseling psychologists, correctional psychologists, school psychologists, psychotherapists, mental health providers from the US Veterans Association, cognitive behavior therapists and Gestalt therapists, and allied mental health professionals (when compared to psychologists). Nurses also made up part of the participant cohort but only when being compared to other professionals in a mental health setting. Study populations included both men and women, while one study had a female sample only (Ben-Zur and Michael, 2007).

There was a lack of papers explicitly reporting the use of theoretical frameworks in the research of burnout amongst this profession, of the job demands and factors that emerged in this review workload and work setting contribute to the burnout experienced by applied psychologists. Age and experience, along with sex were the predominant personal characteristics focused on. We also discuss other resources of interest that emerged from this review below. We delve more deeply into these results in the discussion section of the paper, and present implications for future research.

### Characteristics of burnout measures

All studies sought to examine burnout in their respective professions (see Table 2 for a full list of burnout instruments used). *The Maslach Burnout Inventory–Human Service Scale* (MBI-HSS; Maslach and Jackson, 1996) was the most common form of burnout measurement employed with over a third (34.5%) of papers using this measure. The survey was designed for those who work in human services and the health care sector and covers all three dimensions of burnout (Maslach et al., 2001). The Maslach Burnout Inventory (MBI; Maslach and Jackson, 1996) was also used by an additional third of the papers (31%). All studies examined all three dimensions of burnout, apart from Acker (2012) who utilized the emotional exhaustion subscale of the MBI (Maslach and Jackson, 1996). Work-related burnout and client-related burnout were included in Di Benedetto and Swadling (2014) and D'Souza et al. (2011) studies.

**Table 2:** Burnout measurements used by papers included in review.

**Author(s)**	**Country**	**Sample *N***	**Instruments**
Acker, 2012	USA	460	MBI (EE only)
Ackerley et al., 1988	USA	562	MBI
Ballenger-Browning et al., 2011	USA	97	MBI
Ben-Zur and Michael, 2007	Israel	249	MBI (abbreviated)
Boccio et al., 2016	USA	291	MBI-HSS
Carrola et al., 2016	USA	86	CBI^*^, MBI-HSS
Devilly et al., 2009	Australia	152	CBI
Di Benedetto and Swadling, 2014	Australia	167	CBI
Dreison et al., 2016	USA	358	MBI-HSS
D'Souza et al., 2011	Australia	87	CBI
Emery et al., 2009	Australia	190	MBI - HSS
Garcia et al., 2016	USA	137	MBI-GS
Hardiman and Simmonds, 2013	Australia	89	MBI - HSS
Malinowski, 2013	USA	133	MBI-HSS
McCormack et al., 2015	International	30	Qualitative
Mills and Huebner, 1998	USA	225	MBI
Proctor and Steadman, 2003	USA	63	7 items aligning with previous burnout research
Puig et al., 2012	USA	129	CBI^*^
Rosenberg and Pace, 2006	USA	116	MBI-HSS
Rupert and Kent, 2007	USA	595	MBI-HSS, PBI-R
Rupert and Morgan, 2005	USA	571	PBI-R, MBI
Rupert et al., 2012	USA	595	PBI-R,
Rupert et al., 2009	USA	487/421	MBI-HSS, PBI-R
Rzeszutek and Schier, 2014	Poland	200	OLBI
Senter et al., 2010	USA	203	MBI-HSS
Sim et al., 2016	USA	14	Qualitative
Steel et al., 2015	UK	116	MBI
Viehl and Dispenza, 2015	USA	150	CBI^*^
Vredenburgh et al., 1999	USA	521	MBI

* *Counselor Burnout Inventory*.

### Theoretical framework

Only 12 papers (40%) explicitly stated the theoretical framework under which they conducted their research, see Table 2. Three papers used COR (Hobfoll, 1989), with five using the JD-R model (Demerouti et al., 2001). Other theories utilized include Maslach and Jackson's (1996) model of burnout by Malinowski (2013), Rupert and Kent (2007), and Vredenburgh et al. (1999). Malinowski (2013) also employs the theory of multidimensional humor (Martin et al., 2003). Two papers employ stress models in their studies, Acker (2012) with the role stress theory and Ben-Zur and Michael (2007) who use cognitive model of stress and coping (Lazarus, 1999). Finally, Mills and Huebner (1998) use a transactional model of burnout, personality and situational stressors in which to frame their results.

The remaining papers lacked an explicit reference to the model or theory underpinning their investigation. A theory-driven approach is required to enhance our ability to predict, prevent and understand burnout among psychologists (Rupert and Kent, 2007). A key recommendation from this review therefore is that we need to take a stronger theory driven approach to the study of burnout amongst applied psychologists.

### Emotional exhaustion

Emotional exhaustion emerged as the most commonly reported dimension of burnout, over and above depersonalization and lowered personal accomplishment. In terms of scores from the MBI, this dimension received the highest scores of burnout in over one-third of the papers (34.5%). Mills and Huebner (1998) found that 40% of the psychologists reported high to moderate levels of emotional exhaustion. This parallels the proportion of psychologists (39.9%) that reported feeling high levels of emotional exhaustion in the study conducted by Ackerley et al. (1988). Acker (2012), who only used the emotional exhaustion subscale to investigate burnout, established that more than half (56%) of participants (*N* = 460) reported moderate to high levels of emotional exhaustion, they also found that *emotional* exhaustion has a stronger relationship to the intent to quit than job stress. Psychologists also reported the highest level of emotional exhaustion in comparison to other mental health professionals (Dreison et al., 2016). Employing the development of severity levels approach of Maslach et al. (1986) and Malinowski (2013) reported that those who experienced emotional exhaustion were on the borderline of low to moderate burnout, compared to those who experienced depersonalization or those who had a decrease in personal accomplishment, both of which resulted in lower levels of burnout (Malinowski, 2013). The sample in Rupert and Kent (2007) matched the average or middle range of emotional exhaustion found in psychologists. Furthermore, they credited the occurrence of emotional exhaustion to an individual's total hours worked, administrative work (paper work), negative client behaviors and over involvement with clients, supporting the position that increased workload can have a significant impact on experiential burnout (Maslach et al., 2001).

### Job demands and job factors

#### Workload

Workload and perceived time pressure emerged as the most significant job demand contributing to burnout, repeatedly appearing as a cause of burnout in this review. The relationship between burnout (especially emotional exhaustion) and workload is strong and consistent. Workload contributes to emotional exhaustion by simply placing excessive demands on an individual, exhausting their energies (Maslach et al., 2001). It is not only the objective amount of work that increases workload, the perceived wrong type of work (e.g., administrative work) which is viewed as additional to an individual's role, or if the individual lacks the necessary skills to execute the work, can increase workload.

Ben-Zur and Michael (2007) found a positive correlation between high burnout and the appraisal of stress/load, contributing to the position that appraisal of workload is as impactful as actual load placed on an individual. School psychologists who served multiple schools experienced a higher level of burnout in comparison to those who served single schools (Proctor and Steadman, 2003). The number of hours worked (Rupert and Morgan, 2005), high work demands and a lack of autonomy (Steel et al., 2015), and concerns over having more clinical work than could be accomplished (Garcia et al., 2016) can all lead to or predict higher levels of emotional exhaustion. More work hours per week can also lead to more extensive work-family conflict, which in turn, is related to increased emotional exhaustion at work (Rupert et al., 2009).

Workload was also found to have an impact on other dimensions of burnout. For instance, Ballenger-Browning et al. (2011), in a study of military mental health providers, reported that psychologists who had a greater number of patients per week, had decreased feelings of personal accomplishment. Increased time spent on administrative work, or paper work can also lead to increased emotional exhaustion and decreased personal accomplishment (Rupert and Kent, 2007). Non-clinical tasks and crisis work can be credited as inducing burnout, supporting the concept that it might not only be the amount of work that contributes to burnout, but also work that is additional or external to the role of the psychologist (Sim et al., 2016).

#### Work setting differences

The review found that work setting can have both a positive or negative impact on feelings of burnout when gender is taken into account. When gender is controlled for, work setting does not have as large an effect on burnout. For example, both Di Benedetto and Swadling (2014) and Rosenberg and Pace (2006) found no significant effects for work setting on burnout. This is inconsistent with prior research which indicated that working in a private setting could decrease feelings of general stress, emotional exhaustion and depersonalization, and increase feelings of accomplishment, overall decreasing feelings of burnout (Ackerley et al., 1988; Vredenburgh et al., 1999; Rupert et al., 2009).

In the studies that looked at situational demographics, it was found that working in the private sector, having control over clients, hours worked, and case variability had a positive impact on levels of burnout experienced. Those in the private sector experienced lower levels of burnout than those working in the non-private sector (Ackerley et al., 1988; Vredenburgh et al., 1999; Rosenberg and Pace, 2006). Autonomy is thought to be one of the main contributors to this phenomenon, with those working in the private sector more likely to feel they are having an impact on their own personal accomplishments by treating clients that add directly to their own income (Vredenburgh et al., 1999). Autonomy is also negatively related to burnout in therapists (Steel et al., 2015). Rosenberg and Pace (2006) found that those who worked in the private sector had greater feelings of personal accomplishment in comparison to those who worked in the medical sector and those who worked in academia. It is also thought that the lower levels of work stress experienced by people in the private sector is due to the lack of administration that comes with working in larger organizations. However, Di Benedetto and Swadling (2014) have shown that despite a higher proportion of younger professionals found in non-private practice workplaces, this had no significant effect on any of the dimensions of burnout among Australian psychologists. Rosenberg and Pace (2006), as previously stated, found that those working in private practice settings fared better than their peers on levels of experienced burnout, however they also found a relationship between hours worked and experiential burnout. Such as that when hours worked increased, so did feelings of depersonalization and emotional exhaustion, with feelings of personal accomplishment decreasing (Rosenberg and Pace, 2006).

Somewhat counterintuitively, it has also been found that an increased number of cases or a larger amount of time spent on delivering psychotherapy or intervention can be related to increased feelings of personal accomplishment (Rupert and Kent, 2007). This comes with a caveat, as there is potential for the opposite to occur depending on the type of client and, on the psychologist themselves (Rupert et al., 2015). Ballenger-Browning et al. (2011) found that diagnosing patients with personality disorder is related to depersonalization. Malingering clients also contributed to feelings of cynicism (Garcia et al., 2016). Depersonalization has potential to occur when dealing with clients who exhibit negative or stressful behaviors (Rupert and Kent, 2007). This is because dealing with negative behaviors (e.g., aggressive or threatening behaviors, suicidal threats or gestures, limit testing and psychotic behaviors) requires a larger commitment of emotional energy and time (Rupert et al., 2015).

A burnout risk that is potentially unique to psychologists is over-involvement with clients. For a therapeutic relationship to be successful, the psychologist has to be invested in the client; the risk comes when an over-involved psychologist dedicates an increased amount of emotional energy, thus depleting a valuable resource. Studies have recognized that over involvement with clients is directly linked to both emotional exhaustion and depersonalization (Raquepaw and Miller, 1989; Huebner, 1994). However, other studies show that over involvement can lead to an increase in personal accomplishment (Rupert and Morgan, 2005; Rupert and Kent, 2007). Over involvement had the strongest relationship with emotional exhaustion (*r* = 0.37, *p* = 0.001) in comparison to other antecedents of burnout (i.e., job stress, control, job support, and professional identity) (Lee et al., 2011).

### Job characteristics and personal characteristics

#### Age and experience of psychologists

Age emerged as a consistently studied factor in the research of burnout amongst applied psychologists. This review found that an increase in age is related to a decrease in reported burnout. Younger psychologists in a study by Ackerley et al. (1988), reported higher levels of burnout than their older peers. They posited that with age, psychologists learn to conserve their emotional energy so that it is not depleted. The idea that younger individuals are more susceptible to burnout is supported in subsequent research. Age was able to account for 8.4% of reports of emotional exhaustion and 9.4% of the variance in depersonalization in counseling psychologists conducted by Vredenburgh et al. (1999). Older psychologists also reported lower levels of emotional exhaustion and depersonalization of clients and experienced significantly lower levels of client-related, work-related and personal-burnout in subsequent research by D'Souza et al. (2011) and Rupert and Morgan (2005). For Hardiman and Simmonds (2013) age was found to be the only demographic that could distinguish between high and low burnout. Steel et al. (2015) concluded that it was age and not experience that protected from depersonalization, meaning that it is life rather than years of practice, that mediates cynicism.

#### Sex differences

Whilst commonly included in demographic variables of study samples encompassed in this review, there are mixed results for the relationship between sex or gender differences and burnout amongst applied psychologists. Sex or gender has been credited as a correlate for burnout and staff turnover (Paris and Hoge, 2010). Previous research showed that there were significant differences between males and females and their experienced burnout (see Freudenberger, 1974). However, Ackerley et al. (1988) found no support for women experiencing more burnout than their male peers and colleagues. The relationship between burnout and gender is not a simple one. For example, research has found that men experience more cynicism than women, but women experience more emotional exhaustion than men (Maslach et al., 2001). The finding that male psychologists experience greater depersonalization than their female counterparts was supported in this review (Vredenburgh et al., 1999; Rosenberg and Pace, 2006; Rupert and Kent, 2007) and are more likely to depersonalize their clients (Rupert et al., 2009). Emotional exhaustion was more significantly related to being female, especially when consolidated with work setting, personal resources and therapists' belief (Emery et al., 2009).

Work setting also has consequences for sex differences in burnout experienced. Women experience more emotional exhaustion in agency settings (i.e., working in public health care systems or hospitals), in comparison to those who work in private settings (Rupert and Morgan, 2005; Rupert and Kent, 2007). The results on males' burnout and work setting is inconclusive, in some instances, males' burnout did not differ significantly depending on work setting (Rupert and Kent, 2007). Other research found that male psychologists can experience a greater level of emotional exhaustion, especially in group settings (Rupert and Morgan, 2005). Within a military setting Ballenger-Browning et al. (2011) also found that hours worked and sex resulted in different experiences of burnout, with female therapists experiencing higher levels of emotional exhaustion. Sexual minority males reported greater scores on the CBI than both heterosexual men and sexual minority women (Viehl and Dispenza, 2015).

#### Other resources

Despite evidence pointing to the importance of resources in managing or maintaining work-based well-being, not a lot of research to date has examined the resources of psychologists (Rupert et al., 2015). In this review, it was thought pertinent to consider the influence other resources have on the burnout among applied psychologists. An increased level of personal resources have been shown to reduce emotional exhaustion and increase feelings of personal accomplishment (Emery et al., 2009). In the case of work-based resources, control has received the most attention (Ackerley et al., 1988; Rupert and Morgan, 2005; Rupert and Kent, 2007; Rupert et al., 2009). These studies have shown that there is a significant relationship between all three dimensions of burnout and control, with a greater amount on control having a negative effect on burnout; this is supported by a meta-analysis conducted by Lee et al. (2011). However, the concept of control has not always been defined (Rupert et al., 2015) and therefore it is hard to make a definitive assumption on what is perceived as control in the context of burnout.

Workplace support has also been investigated in the research of burnout in psychologists and mental health professionals. Ben-Zur and Michael (2007) found that co-worker support had a negative effect on burnout, and was related to lower levels of emotional exhaustion, depersonalization and higher levels of personal accomplishment. Rupert and Kent (2007) found that those who work in solo private practices experienced lower levels of social support. McCormack et al. (2015) found that work based social support was more effective at reducing burnout than external sources of social support. Although the support required or experienced by a psychologist may depend on their own work setting, experience and, level of training, the consensus appears to be that support is a significant resource for those in the field of mental health (Rupert et al., 2015). Indeed, the results of a meta-analysis show that workplace support is the only resource that may have a significant relationship with personal accomplishment (Lee et al., 2011).

Dreison et al. (2016), who used the JD-R as their guiding theoretical framework, found that education was related to lower levels of depersonalization and higher levels of personal accomplishment. Other resources such as supervisory autonomy support and self-efficacy predicted significantly lower emotional exhaustion. Self-efficacy also significantly predicted higher levels of personal accomplishment. Job resources (i.e., supervisor autonomy support, self-efficacy, and staff cohesion) were all negatively correlated to emotional exhaustion and depersonalization. The qualitative study conducted by Sim et al. (2016), which also employed the JD-R found that the following resources: support from colleagues and the director; the sense that staff work well together; the presence of autonomy; respect for setting limits at work; and social activities with colleagues during the workday all contributed to decreased feelings of burnout. These studies contribute to the argument that the burnout and well-being of psychologists needs to be studied more rigorously using strong theoretical frameworks to guide the research.

One study examined the use of personal resources such as recreational activities, self-care activities, social support and cognitive coping skills and found that an increase in personal resources was related to lower levels of reported burnout, with decreased emotional exhaustion and increased personal accomplishment (Emery et al., 2009). Humor was also found to be a beneficial resource in decreasing feelings burnout with adaptive forms of humor being more beneficial in decreasing feelings of burnout than maladaptive forms (Malinowski, 2013). Interestingly, Puig et al. (2012) found that burnout was negatively related to an individual's exercise and nutrition; meaning that counsellors do not engage in regular or appropriate exercise and nutrition when they experience job stress induced exhaustion. The direction of this relationship is unclear, as Puig et al. (2012) posit that a lack of appropriate exercise and nutrition may in fact contribute to the manifestation of burnout in counsellors.

Cognitive strategies, such as problem solving and prioritizing work (Emery et al., 2009), gaining self-awareness, adjusting perspectives and even fantasizing about leaving (Sim et al., 2016) are important for keeping a perspective on work, resulting in a reduction in emotional exhaustion and depersonalization. The practice of behavioral strategies (creating boundaries and adjusting work schedules) has been shown to have the same effect (Sim et al., 2016). Maintaining work-life balance may also be valuable for lower levels of emotional exhaustion (Rupert et al., 2015). The use of career-sustaining behaviors and self-care behaviors may play a positive role on positive work attitude.

Despite evidence pointing to the influence of work-family issues on psychologist burnout, work-family conflict can increase emotional exhaustion and depersonalization and decrease personal accomplishment (Rupert et al., 2009).

## Discussion

The intention of this review was to investigate the prevalence and cause(s) burnout of applied psychologists and allied mental health professionals. Although research on burnout was first conducted with those from the caring professions, there is a paucity of articles explicitly examining burnout among applied psychologists. Therefore, the objective of this review was to scrutinize the research literature regarding burnout among this field.

One of the main findings of the review supported the idea that emotional exhaustion is the most commonly reported dimension of burnout. Emotional exhaustion relates to the most basic interpretation of the stress response, and most commonly shows a positive correlation between work demands and stress related health outcomes (Acker, 2012). It most often leads an individual to seek an escape or distance themselves from their work both emotionally and cognitively, and is thought to lead on to feelings of cynicism (Maslach, 2003). The studies that specifically looked at the distinct dimensions of burnout all found emotional exhaustion scores to exceed those in the other burnout dimensions, supporting a review of burnout in clinical psychologists in the UK (Hannigan et al., 2004). The nature of a psychotherapists work requires them to provide a service and to care about the people they work with, this type of work is very demanding and requires high levels of involvement, it is not surprising that emotional exhaustion is such a common response to this kind of job overload (Maslach et al., 2001). A lack of energy, negative affect and the perception that emotional resources have been drained can all characterize the feelings of emotional exhaustion (Maslach et al., 1986).

The relationship between high workload and burnout was also supported by this review. The relationship between burnout (especially emotional exhaustion) and workload is strong and consistent (e.g., Rupert and Morgan, 2005; Rupert and Kent, 2007). Workload contributes to emotional exhaustion by placing excessive demands on an individual, and exhausting their energies (Maslach et al., 2001). It is not only the objective amount of work that increases workload, the perceived accessory work (e.g., administrative work) which is viewed as additional to an individual's role, or if the individual lacks the necessary skills to execute the work, can increase workload. Workload mismatch is also pertinent when an individual has to express emotions that do not match their own (Maslach et al., 2001). Previous research demonstrates workload is associated with burnout in nurses (Greenglass et al., 2001; Van Bogaert et al., 2014); physicians (Nishimura et al., 2014); and teachers (Van Droogenbroeck et al., 2014), thus exemplifying the diversity in research and populations showing that it is a prevalent cause of burnout regardless of career or nationality.

The importance of social support in managing stress and burnout is well documented (Hobfoll, 1989; Halbesleben, 2006). Social support, work-based support and supervisor support have all been credited as a resource for reducing burnout in clinical psychologists (Hammond et al., 2017). Franco (2015) suggests that supervisors educate themselves and form open discussions with their clinicians, promoting self-awareness and self-care practices amongst those they supervise. However, it is not a requirement for all disciplines of applied psychologists to have specialist supervisory or peer-based support, e.g., applied sport psychologists. Yet these professionals often face more than just performance issues (McCann, 2008; Elsborg et al., 2015), and can go through experiences that fluctuate from intense, almost euphoric, positive emotion to acute negative emotion, whilst having to maintain their role as a practitioner who provides a solid source of support for others, without expressing their own emotions. As previously stated in this review, burnout can often occur when an individual has to exhibit one emotion whilst experiencing another conflicting emotion (Maslach et al., 2001). Rupert and Morgan (2005) recognize that the cost of burnout to a psychologist can be high, the distress being experienced by the individual suffering from burnout is only one consequence, there is also the potential harm to the client who is receiving services from a psychologist operating at a diminished capacity and not to mention the turnover costs for employees. It is also thought that those entering the profession of psychology, are more susceptible to suffering from mental health issues as the attraction to the industry lends itself to the practitioners need to understand their own problems (Bearse et al., 2013). Psychologists are potentially predisposed to burnout through putting others needs before their own and despite the knowledge that social support can buffer against the negative effects of burnout, many psychologists themselves are reluctant to seek their own professional help (Bearse et al., 2013). Bearse et al.'s investigation found that one of the barriers to psychologists seeking professional help is the idea that the professional help is hard to find. Worryingly, almost 60% of respondents claimed that they did not seek help even though they were aware that they could have benefitted from it at the time (Bearse et al., 2013).

A concerning finding from this review was the lack of theoretically driven research. It has been previously noted that there is a lack of comprehensive theory driven research into psychologists' burnout (Rupert and Kent, 2007), especially that which is related to demands and resources. In other sectors, such as organizational psychology, investigations into the phenomenon of burnout utilize various models to explain its occurrence (see Chirico, 2016). The majority of papers included in this review did not explicitly employ a theoretical framework see Table 2 for further information. Of the 12 papers that did employ a theoretical framework, the main ones used by papers in this review were COR theory (Hobfoll, 1989) or the JD-R model (Bakker et al., 2014). While research into resources and their effect on burnout in psychologists has been conducted, it is not extensive enough to be in line with mainstream research on burnout. In accordance with COR theory where an individual will strive to create, maintain and build on their resources (Hobfoll, 1989), burnout is the result of a slow loss of resources brought about by continual stress from the work environment. Therefore, despite emotional exhaustion being the most commonly experienced dimension of burnout, it would not be surprising if that trend continued for a practitioner, and the other dimensions of burnout should soon follow (Gorgievski and Hobfoll, 2008). A review by Rupert et al. (2015) sought to consolidate the research into what resources effect burnout amongst psychologists, yet they found that different resources were examined in different ways. Indeed, as suggested by the authors, in order to gain “a complete understanding of the emergence of burnout, it is important to investigate the multiplicative impact of demands and resources” (Bakker et al., 2005, p. 178). Interestingly, Crawford et al. (2010) suggest a “differentiated job demand-resource model” (p. 843) that expands the JD-R to distinguish the interpretation of job demands as either challenging or as a hindrance, as this could have a differential effect on burnout. While certain demands can be viewed as debilitating for some, others may view them as an opportunity to expand their repertoire. In a similar way to creating burnout intervention specific to the individual, research into resources effects on burnout could be done the same way. Once this is achieved, a clearer and more concise understanding of burnout in applied psychologists can be realized.

Demographics such as age and gender were also revealed as popular characteristics used to predict burnout in this review. Age was used consistently as a personal characteristic to predict burnout among applied psychologists, with age being negatively associated with burnout. Although there is a trend for younger psychologists to experience higher levels of burnout, it is believed that burnout can result in career termination and so therefore one could speculate that as psychologists mature, not only are they more able to cope with work stressors, it could be that those more susceptible to burnout end up leaving the profession.

It is clear from our review that there are conflicting views on the prevalence of burnout and its causes in relation to the sex of the individual. The findings among psychologists echo the results found by Purvanova and Muros (2010) on burnout and gender. Results from their meta-analysis of 183 studies challenged the belief that women were more prone to burnout than men were (Purvanova and Muros, 2010). Results showed that women experience more emotional exhaustion than men do (mean effect size = 0.10), whereas men experience more depersonalization than women do (mean effect size = −0.19). These findings state important implications on how to manage and prevent burnout for different populations, potentially specifying interventions depending on sex (Purvanova and Muros, 2010). Our review shows that the prevention and management of burnout in psychologists should be approached differently not only depending on sex, but also in relation to work-setting, and sexual identity. Interventions should therefore be designed to fit the work setting, gender identity and even sexual orientation. This could require further in-depth research into sex, work setting, sexuality and their combined effect on burnout.

Despite the awareness of the importance of minding one's own mental health and armed with the knowledge of how to do so, many applied psychologists appear not to practice what they preach. Progress in reducing the stigma surrounding the application of psychological interventions has been made; however, it may be time to ensure that those delivering psychological services are heeding the same message.

### Future research

Longitudinal studies, which examine the staying power of burnout, have been utilized with other professions (see Hakanen and Schaufeli, 2012), however, the long-term effects of burnout in applied psychologists has not been investigated. The absence of research that investigates the long-term effects of burnout in applied psychologists is of concern. It is our duty, after all, to safeguard ourselves from the ill effects of burnout, not only for our personal well-being but for the well-being of those in our care.

According to the review by Hannigan et al. (2004), multiple sources contribute to stress of clinical psychologists in the UK. These included client characteristics, excessive workloads, professional self-doubt and poor management. By expanding the demographics, this review added to these sources with private practice vs. public health providers, work hours, work-setting, age and length of experience, gender, sexual orientation, resources and other factors. By considering studies from the US, Europe and Australia, we are able to glimpse a larger international perspective on the prevalence of burnout among practicing psychologists. However, more extensive analysis needs to occur in order to examine potential cultural differences or see if the demands of the profession transcends culture.

Tools to combat the initiation of burnout amongst psychologists have also been under-researched. Mindfulness has been recognized as a useful resource to use in the treatment and prevention of burnout (Di Benedetto and Swadling, 2014). Research showed a strong negative correlation between the practice of mindfulness and levels of burnout experienced (Di Benedetto and Swadling, 2014). Non-reactivity to inner experience, acting with awareness, describing and non-judging of inner experience were the four facets of mindfulness that were significantly negatively correlated with burnout. This suggesting that mindfulness based interventions may be a useful preventative strategy for practicing psychologists, and would benefit from further research with this specific population. Rees et al. (2015) have developed a model which suggests that psychological resilience will mediate relationships between workplace stress and variables such as coping and adjustment, but requires further research especially with those in the caring professions.

A novel nature-based intervention approach is another route to prevention using the principles of psychological recovery (Bloomfield, 2017). Coupling environmental cues, positive environmental stimuli (e.g., good air quality) with a sense of detachment may offer a remedy for workplace well-being (Sonnentag, 2012). For examples, Steidle et al. (2017) demonstrated that savoring nature and progression muscle relaxation interventions could enhance energetic resources (vigor) on both a daily and longer-term basis in employees. As vigor is a component of work engagement, this could be a simple, yet useful, intervention to maintain energy resources on a daily basis. Clauss et al. (2018) have also showed that employing a positive work reflection intervention, focusing on fostering personal resources can reduce emotional exhaustion and fatigue in caregivers. An approach which could yield similar results in this caring profession.

Ultimately, research regarding the impact of job resources on burnout is more extensive than the impact of personal resources on burnout. From a personal resource point of view, there seems to be even less investigation into what can assist in the treatment and prevention of burnout in psychologists. There is a dearth of knowledge regarding other resources that may reduce the risk of burnout in psychologists, such as savoring, recovery, resilience, opportunities for development and, job crafting.

## Conclusion

The aim of this review was to synthesize research evidence for the prevalence and cause(s) of burnout among applied psychologists and allied mental health professionals. In this review we addressed the gap in the research literature on practitioner burnout. Applied psychologists are susceptible to burnout, especially to emotional exhaustion. The research lacks strong theoretical frameworks, although using the COR and JD-R theories show us that workload and work setting are positively related to the burnout experienced by applied psychologists, whilst age, experience and sex can have a negative relationship with burnout. Burnout among psychologists can have a detrimental effect not only on the individual but also to the people receiving their care. Therefore, further research in this area and specific population has implications for the profession at large and the well-being of both practitioners and their clients.

## Author contributions

HM conducted the literature search and analyzed the data. All authors contributed to the conceptualization, design, and interpretation of the data and to drafting and revising of the manuscript. All authors are responsible for the final version of the manuscript and take equal responsibility for results published.

### Conflict of interest statement

The authors declare that the research was conducted in the absence of any commercial or financial relationships that could be construed as a potential conflict of interest.
